# Development, Disease, and Regeneration of Tissues in the Dental-Craniofacial Complex

**DOI:** 10.1155/2013/836871

**Published:** 2013-08-27

**Authors:** Brian L. Foster, Yong-Hee P. Chun, Erica L. Scheller, Zhao Lin, Chad M. Novince, Avina Paranjpe

**Affiliations:** ^1^National Institute of Arthritis and Musculoskeletal and Skin Diseases (NIAMS), National Institutes of Health (NIH), 9000 Rockville Pike Building 50, Room 4120, Bethesda, MD 20892, USA; ^2^Department of Periodontics, University of Texas Health Science Center at San Antonio, TX 78229, USA; ^3^Department of Molecular and Integrative Physiology, University of Michigan Medical School, Ann Arbor, MI 48105, USA; ^4^Department of Periodontics, Virginia Commonwealth University School of Dentistry, Richmond, VA 23298, USA; ^5^Department of Periodontics, University of Washington School of Dentistry, Seattle, WA 98195, USA; ^6^Department of Endodontics, University of Washington School of Dentistry, Seattle, WA 98195, USA

## 1. Introduction

This special issue of BioMed Research International focuses on the theme of development, disease, and regeneration of tissues in the dental-craniofacial complex, highlighting dynamic research that makes this an exciting area of biomedical research. Dental and craniofacial diseases have significant implications for the oral and systemic health of the general public. The dental-craniofacial tissues, more than any other tissue and organ of the body, are key for communication and mastication. We are delighted to present in this special issue of biomed research international a glimpse into diseases of dental and craniofacial tissues, biology in development, and therapeutic innovations. The theme was chosen in order to include fundamental basic science research highlighting normal developmental processes and cases where these go awry (including disease models), as well as translational and clinical papers investigating therapeutic strategies for repair and regeneration of tissues. The aim of this introduction is to highlight central concepts of the 11 individual papers under each theme and further illuminate topics to be found in the papers and throughout the special issue.

## 2. Disease

Diseases of the dental-craniofacial complex include a broad spectrum of illnesses, ranging from rare hereditary conditions to some of the most prevalent pathologies in the world, including periodontal disease and premature tooth loss. Osteogenesis imperfecta (OI) is a rare inherited skeletal disorder associated with defective collagen structure, production, or processing. Dental defects arising as a result of OI represent one form of dentinogenesis imperfecta (DGI), which include reduced and defective dentin mineralization, alterations in tooth crown and root formation, and predisposition to tooth fractures and abscesses. In their report, A. Boskey et al. employed the Brtl/+ Gly349Cys knock-in mouse as a model for type IV OI, adopting an array of imaging techniques, including the Fourier transformed infrared microscopic imaging (FTIRI), scanning electron microscopy (SEM), and microcomputed tomography (micro-CT) to elucidate the etiology of dentin defects in this mouse. The authors report reduced mineralized dentin volume in molars of the knock-in mice, without changes in enamel. The expected alterations in collagen structure were observed at two months of age but self-corrected by six months. FTIRI identified increased acid phosphate content at both ages in knock-in mice, implying dentin matrix mineralization defects. These results indicate an impaired matrix mineralization and a slower correction of the phenotype with age, implying that both the collagen matrix and the noncollagenous proteins that regulate the function of that matrix may be altered in the *Brtl*/+ teeth and shedding light on pathological mechanisms involved in tooth defects in patients with DGI. 

Nonsyndromic DGI is caused by mutations in the dentin sialophosphoprotein (*DSPP*) gene. DSPP encodes for a large precursor protein that is cleaved into dentin sialoprotein (DSP) and dentin phosphoprotein (DPP), two prominent extracellular matrix components involved with dentin mineralization. In their paper, “*A DSPP mutation causing dentinogenesis imperfecta and characterization of the mutational effect*,” S. K. Lee et al. identified a mutation in exon 2 of *DSPP* in association with type III DGI in a Korean family. The DGI phenotype was quite severe, featuring tooth discoloration, severe attrition, and periapical inflammation. Through mutational analysis and *in vitro* assays, the authors report that the *DSPP* mutation resulted in retention of the mutant protein in the endoplasmic reticulum, leading to defective secretion of DSP. The same mutation was also found recently in a Chinese family and, interestingly, alters the same propeptide cleavage site mutated in the classic Brandywine isolate of type III DGI, adding to genotype-phenotype understanding of DGI and other diseases affecting dentin formation and mineralization. 

Craniosynostosis is a condition in which premature fusion of cranial bones leads to elevated intracranial pressure and dysmorphic cranial and facial shapes. Repeated surgical intervention is typically required to manage the associated morbidity and negative effects on quality of life. The Crouzon syndrome, associated with gain-of-function mutations in fibroblast growth factor receptor 2 (FGFr2), features craniosynostosis. The mechanism of its development remains incompletely understood. J. Liu et al. studied the *Fgfr*2^C342Y^ mouse model of the Crouzon syndrome, documenting that primary osteoblasts from the Crouzon mice feature elevated expression of early osteoblast markers, but reduced alkaline phosphatase and late osteoblast markers, and inhibition of mineralization in 2D and 3D cultures. They also report, for the first time, defective long bone formation in *Fgfr*2^C342Y^ animals. Their findings suggest that development of the axial and appendicular skeleton in *Fgfr*2^C342Y^ mice is impaired due to cell autonomous defects in the osteoblast.

Periodontal disease, affecting the tooth root cementum, periodontal ligament, and surrounding alveolar bone, affects almost 50% of US adults and, worldwide, is the most prevalent cause of premature tooth loss. Despite numerous animal models focusing on periodontal disease progression, few studies have assessed the role of functional mechanics of the bone-PDL-tooth joint in disease progression. J. Lee et al. take this approach by employing a lipopolysaccharide soaked ligature induced rat model of periodontal disease. The authors mapped two trends in response to disease induction. First, inflammation-induced degeneration of more coronal root tissues and, second, mechanobiological changes in the apical periodontal regions, potentially resulting from coronal degeneration over time. Interestingly, the authors noted that coronal induction of inflammation by ligature placement affected the overall distribution of proinflammatory cytokine TNF-*α* in the entire periodontal complex. Breakdown and compromise of coronal attachment shifted physiological function into an impaired function mode that in turn led to accelerated tissue adaptation to meet functional demands, including increased secondary cementum formation. Overall, this study adds to the understanding of periodontal disease progression and prompts future studies to consider biomechanical changes in addition to biochemical and morphological changes in periodontal disease and other cases of joint inflammation. 

## 3. Development

Craniofacial development encompasses numerous pre- and postnatal processes, including growth of the cranium by both endochondral and intramembranous ossification, development of soft tissues including salivary glands, tongue, and the musculature, and odontogenesis, wherein two separate sets of teeth form in humans (the primary and secondary dentition), with each tooth home to three distinct mineralized tissues, the enamel, dentin, and cementum. The morphogenesis and growth of dental and craniofacial tissues is influenced by neural crest cells, cell-cell communications, and environmental factors. 

Proper development of the mandibular cartilage is important for growth and mineralization of the mandible. However, while endochondral ossification of the axial and appendicular skeletal elements is well studied, the mandibular cartilages, which derive from ectomesenchyme of the first pharyngeal arch, are often overlooked or assumed to operate under a similar differentiation program as cartilage with different origins. In their paper, “*An immunohistochemistry study of Sox9, Runx2, and Osterix expression in the mandibular cartilages of newborn mouse*,” H. Zhang et al. investigated the developmental expression pattern of three key transcription factors, Sox9, Runx2, and Osterix, in cartilages of the mouse mandible. Interestingly, the authors report an overlapping expression pattern for Sox9 and Runx2 in mandible that is distinct from limb bud cartilage. Furthermore, despite similar localization of Osterix in mandibular secondary cartilages compared to limb bud, an intense expression of this factor in the degrading portion of Meckel's cartilage of the mandible suggests a role in the ongoing processes there, possibly even phenotypic conversion of these chondrocytes. Overall, these data provide valuable information on an understudied aspect of mandibular development, providing insights for disease mechanisms in conditions such as agnathia and micrognathia, for example. 

The reorientation and fusing of the palatal shelves are critical steps in formation of the secondary palate, where developmental defects can lead to cleft palate or cleft lip and palate. It has been hypothesized that changes in extracellular matrix (ECM) are involved in raising of the palatal shelves to the horizontal position and, further, that epithelial-mesenchymal interactions may be involved in the fusion of the palatal shelves. A. Hirata et al., in their paper, investigated the developmental expression of heparanase and its substrate, heparin sulfate, in the ECM during palate formation in mice. These authors documented heparanase expression in the medial epithelial seam in palatal shelves in the process of fusing, in parallel with expression of additional ECM remodeling enzymes, matrix metalloproteinases 2, 3, and 9, where expression of heparin sulfate, perlecan, laminin, and collagen type IV was depleted. The distribution of this panel of ECM enzymes is consistent with the hypothesis that they play a role in palatal ECM remodeling and proper fusion of the hard palate.

Salivary gland function is essential not only for eating but also for speaking and prevention of dental decay. Reduced salivary gland function can result from diseases such as Sjögren's Syndrome and as a result of therapeutic radiation for treatment of head and neck cancers. Great strides have been made in understanding salivary gland function, and a phase 1 clinical trial currently underway at the National Institute of Dental and Craniofacial Research (NIDCR) is evaluating the potential for gene therapy using water channel Aquaporin 1 (AQP1) to improve salivary flow in patients with radiation-induced damage to parotid gland function (ClinicalTrials.gov NCT00372320). While salivary gland epithelial cell differentiation and branching have been the subject of intense study, the mesenchymal cell component is less well understood. K. Janebodin et al. employed a transgenic reporter mouse to study submandibular salivary gland mesenchyme *in vivo* and *in vitro*, developing an approach to study epithelial-mesenchymal interactions *in vitro* in order to better understand how signals such as TGF-*β*1 affect cell differentiation and formation of the functional acinus.

Teeth also form by crosstalk between the specialized odontogenic epithelium and ectomesenchyme arising from migrating cranial neural crest cells. The ameloblasts, highly specialized secretory cells derived from the epithelium, synthesize the enamel of the tooth crown, a unique epithelial mineralized tissue that is the hardest tissue in the human body. H. Li et al. studied the role of multifunctional membrane protein NUMB in a variety of dental cells, including ameloblasts, odontoblasts, and dental pulp cells, and found that interaction of this factor with the notch signaling pathway may have regulatory effects on ameloblast differentiation “*Expression and function of NUMB in odontogenesis*”. Greater understanding of ameloblast differentiation is critical for understanding hereditary enamel defects, as well as efforts towards regenerating enamel or bioengineering teeth from dental stem cells, as the enamel organ is completely lost upon eruption of teeth, leaving no known progenitor cells behind. 

Enamelin is a component of the enamel extracellular matrix, and mutations in the associated *ENAM* gene cause autosomal dominant amelogenesis imperfecta, marked by thin or pitted, easily abraded enamel and, in some cases, enamel aplasia. Lower body weight in *Enam* null mice prompted A. H.-L. Chan et al. to study the potential for enamel defects to influence body weight, operating with the hypothesis that dental pain arising from defective tooth structure causes reduced nutritional intake. The authors discovered that the significantly decreased average litter body weight of *Enam* null mice was improved by implementing a soft chow diet over the first six weeks of life. Because enamelin is ameloblast specific, this effect on body weight is likely occurring via the interaction between the defective enamel phenotype and reduced ability to feed on a hard diet. The fact that food hardness becomes an important variable in body weight gain in mice with defective dentition can provide some mechanistic insight into the relationship between compromised oral health and poor growth in children, where dental caries and dental pain and sensitivity may contribute to poor nutrition. 

## 4. Regeneration

Despite a wide range of approaches, periodontal therapies are currently unpredictable, few are truly regenerative, and many lack a biologic foundation. Research has shown that periodontal disease can be successfully treated, with biological periodontal tissue regeneration (i.e., return to structure and function of cementum, PDL, and bone) in some cases. Q. Li et al. focus on platelet-rich fibrin (PRF) as a potential scaffold for periodontal regeneration. The PRF technique was developed from platelet-rich plasma (PRP) which has been used to promote healing and regenerate bone and periodontal tissues. For PRP, platelets are concentrated and activated with thrombin and calcium chloride to promote instant release of growth factors and cytokines. In contrast, PRF is not activated by thrombin or anticoagulants and releases growth factors gradually. *In vitro*, PRF increased markers of proliferation and differentiation in periodontal progenitor cells, and isolated use of the major PRF component, fibrin, replicated these effects. Further, *in vivo* studies in mice supported that PRF promoted integration and soft tissue healing, and pilot studies placing PRF in peri-implant locations in two human patients were associated with new alveolar bone formation. 

Finally, in their review article, T. A. El-Bialy and Alhadlaq create a primer on therapeutic approaches for underdeveloped mandibles, covering bite-jumping (functional) appliances, low intensity pulsed ultrasound (LIPUS), hormone treatment, photobiomodulation, and gene therapy. While these techniques hold promise for improving health outcomes for children with mandibular growth deficiency, all have inherent limitations and challenges, as detailed by the authors. 

## 5. Concluding Remarks

This special issue of Biomed Research International showcases a unique collection of 11 papers that serve as touchstones for several areas of dental-craniofacial research for like-minded researchers, clinicians, and students, as well as scientists in other disciplines. These papers touch many of the exciting aspects of these fields in recent years, and we hope they will stimulate thought and further research to better understand these aspects of human health. Researchers and clinicians have never been better positioned to understand mechanisms of development, disease, and regeneration of dental and craniofacial tissues and, in turn, to apply that knowledge to make clinically meaningful advances.

*Brian L. Foster* *Yong-Hee P. Chun* *Erica L. Scheller* *Zhao Lin* *Chad M. Novince* *Avina Paranjpe*

